# Molecular dynamics simulations of membrane proteins and their interactions: from nanoscale to mesoscale

**DOI:** 10.1016/j.sbi.2016.06.007

**Published:** 2016-10

**Authors:** Matthieu Chavent, Anna L Duncan, Mark SP Sansom

**Affiliations:** Department of Biochemistry, University of Oxford, South Parks Road, Oxford OX1 3QU, UK

## Abstract

Molecular dynamics simulations provide a computational tool to probe membrane proteins and systems at length scales ranging from nanometers to close to a micrometer, and on microsecond timescales. All atom and coarse-grained simulations may be used to explore in detail the interactions of membrane proteins and specific lipids, yielding predictions of lipid binding sites in good agreement with available structural data. Building on the success of protein–lipid interaction simulations, larger scale simulations reveal crowding and clustering of proteins, resulting in slow and anomalous diffusional dynamics, within realistic models of cell membranes. Current methods allow near atomic resolution simulations of small membrane organelles, and of enveloped viruses to be performed, revealing key aspects of their structure and functionally important dynamics.

## Highlights

• Simulations provide a computational tool to probe membrane structure and dynamics.
• Simulations can successfully predict lipid binding sites on membrane proteins.
• Large scale simulations reveal crowding and clustering of proteins in membranes.
• Near atomic resolution models of organelles and enveloped viruses are now possible.

## Graphical abstract

**Figure:**
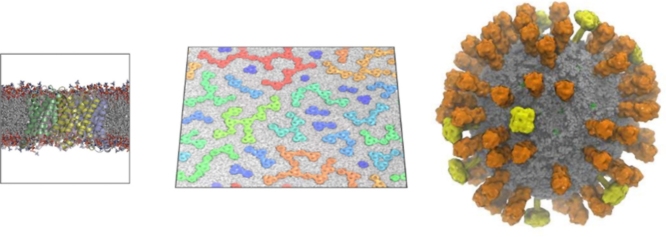

**Current Opinion in Structural Biology** 2016, **40**:8–16

This review comes from a themed issue on **Biophysical and molecular biological methods**

Edited by **Petra Fromme** and **Andrej Sali**

For a complete overview see the Issue and the Editorial

Available online 21st June 2016

**http://dx.doi.org/10.1016/j.sbi.2016.06.007**

0959-440X/© 2016 The Authors. Published by Elsevier Ltd. This is an open access article under the CC BY license (http://creativecommons.org/licenses/by/4.0/).

## Introduction

Membrane proteins play a key role in the biology of cells. Around 20% of genes encode membrane proteins, and they form a major class of drug targets. There has been considerable progress in the structural biology of membrane proteins resulting in over 2500 structures in the PDB, corresponding to over 700 distinct membrane protein species [1^•^]. Molecular dynamics (MD) and related molecular simulation approaches provide important tools which allow us to simulate both individual membrane proteins and more complex membrane systems [2]. Thus, MD simulations have become a valuable addition to the range of experimental structural and biophysical techniques for studying membrane proteins and their interactions with lipids [3].

**Figure 1:**
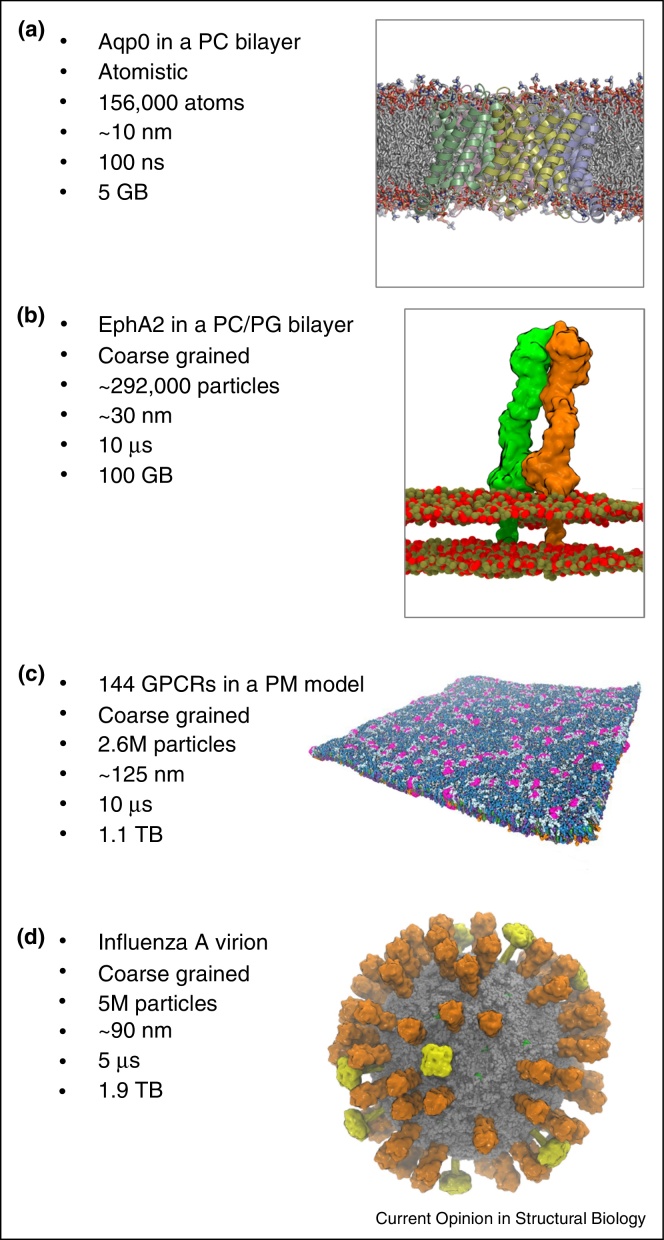
Overview of MD simulations of membranes. For each simulation granularity of the simulation (atomistic versus coarse-grained), the number of atoms/particles (including water, which are omitted for clarity from all of the images) in the simulation system, the approximate linear dimension of the simulation box, the duration of the production run simulation, and the resultant trajectory file size are given. **(a)** A single integral membrane protein (Apq0) in a phospholipid bilayer [10] (figure courtesy of Dr. Phillip J Stansfeld). **(b)** CG simulation of an EphA2 receptor dimer [21], with the lipids in brown (PC) and red (PG). Reprinted with permission from [21]. **(c)** A large plasma membrane (PM) model containing multiple copies of a GPCR. Seven different lipid species are present in an asymmetric bilayer (blues/grey/green/orange), with the GPCRs (S1P1 receptors) in pink. Reprinted with permission from [51^•^]. Copyright 2015 American Chemical Society. **(d)** The membrane envelope of a complete influenza A virion with the lipids in grey, hemagglutinin in orange, neuraminidase in yellow and the M2 channel protein in green [65^••^] (figure courtesy of Dr. Tyler Reddy).

In this article we will review two major and complementary trends in molecular simulations of membrane proteins: (i) to probe protein–lipid interactions of single membrane proteins and (ii) to model more complex membranes containing mixtures of multiple lipid species and multiple copies of membrane proteins (Figure 1). It remains a challenge to develop biologically realistic models of cell membranes, but recent methodological advances enable an integrated approach to the problem, drawing together structural, biophysical and biochemical data into dynamic models which aid interpretation of structural and imaging data on membranes of cells and their organelles. We will survey these advances and a number of recent applications. We will therefore also discuss the development of mesoscale approaches which allow very large scale simulations, exploring membrane behaviour beyond the nanoscale and thus narrowing the gap between simulations and experiment.

## Lipid–protein interactions at the nanoscale

MD simulations may be thought of as a computational microscope [4]: one may ‘zoom in’ to atomic resolution to examine detailed interactions of a membrane protein with water, ions, and lipids, or ‘zoom out’ to a lower resolution using for example coarse-grained (CG) [5••, 6] simulations to address longer length and timescales, albeit with some loss of detail in modelling interatomic interactions. This approach has been successfully used to reveal the dynamic interactions of membrane proteins with lipids at the nanoscale [1•, 7, 8].

Simulations have been used to predict lipid interaction sites for a number of mammalian integral membrane proteins, providing detailed views of both the lipid annulus, as for aquaporin [9, 10] (Figure 1a), and of interactions of specific lipids. A number of recent studies have characterised experimentally observed interactions between cholesterol molecules and G-protein coupled receptors (GPCRs), reviewed in [7], and have explored how such protein–lipid interactions may modulate the dimerization of GPCRs and its possible effects on receptor function (see e.g. [11]). In addition to interactions of membrane proteins with cholesterol, simulations have been used to identify binding sites for phosphatidyl inositol 4,5-bisphosphate (PIP_2_) binding sites on ion channels, transporters, and receptor proteins. Thus, PIP_2_ binding sites have been characterised for ion channels including Kir2.2 [12] and Kv7.1 [13] potassium channels, and PIP_2_ regulation of dopamine transporters has been explored [14^•^]. CG simulations have been used to compare interactions of PIP_2_ molecules with the transmembrane and juxtamembrane domains of all 58 human receptor tyrosine kinases [15^•^], illustrating how high throughput approaches to membrane protein simulations [16, 17] enable systematic surveys of families of membrane proteins and their lipid interactions. CG simulations have also been used to explore the free energy landscapes of PIP_2_ and of glycolipids with the transmembrane domain of the EGFR [18].

More recent simulation studies of intact receptor tyrosine kinases (i.e. not simply the transmembrane domain) have revealed how lipid mediated interactions between the membrane surface and the ectodomains of the receptor may modulate the overall conformation of these complex multi-domain membrane proteins. Thus ectodomain/bilayer interactions may result in an asymmetric conformation of the EGFR dimer [19^•^], and these interactions can be influenced by receptor glycosylation [20^••^]. Ectodomain/bilayer interactions of the related EphA2 receptor are mediated primarily via anionic lipids, and may stabilize different conformations at the membrane of liganded versus unliganded forms of the receptor [21] (Figure 1b).

**Figure 2:**
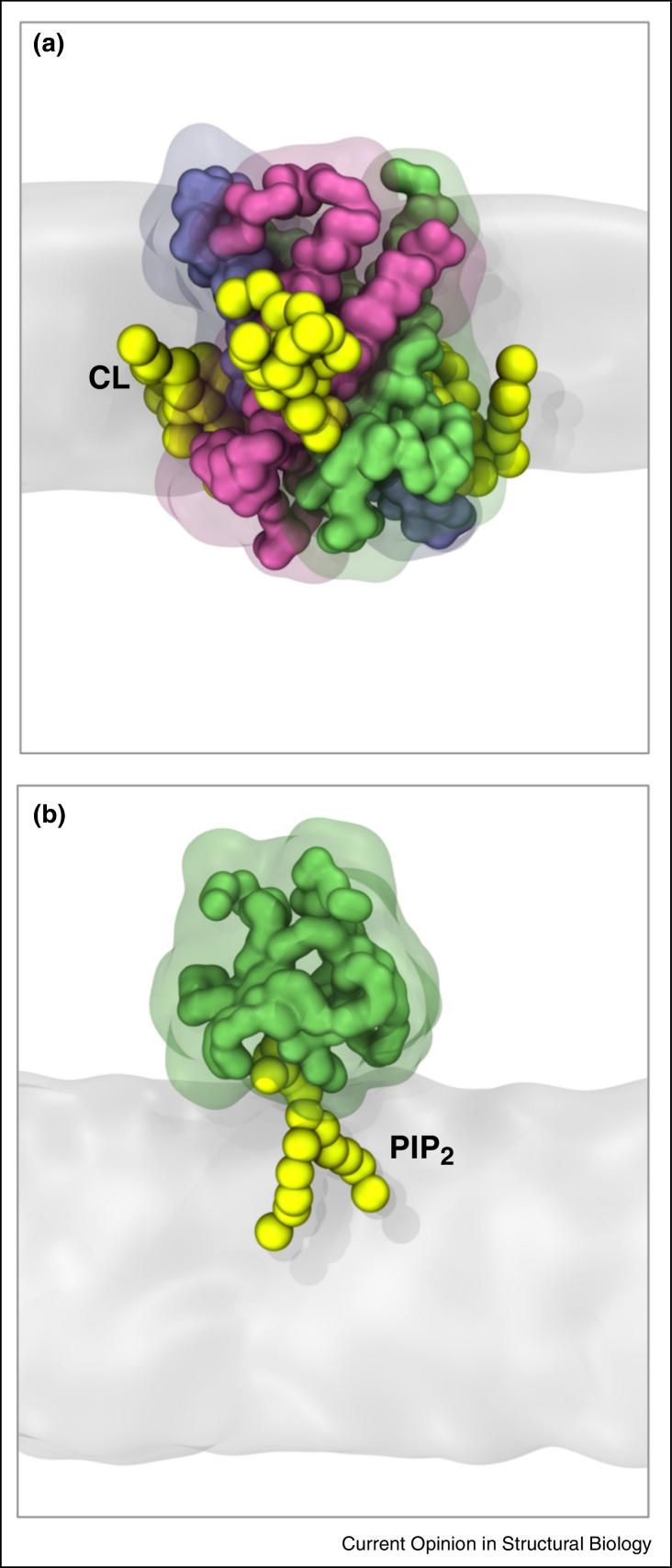
Protein–lipid interactions via coarse-grained simulations. **(a)** The mitochondrial ADP/ATP carrier ANT1 (with the three domains in green, pink and blue) interacting with three cardiolipin molecules in yellow. The lipid bilayer is shown in grey [7]. **(b)** A GRP1 PH domain (green) at the surface of a lipid bilayer bound to a PIP_2_ molecule (green) [35^••^] (figures courtesy of George Hedger).

Simulations have also been used to explore the interactions with proteins of more ‘specialized’ lipids from mitochondrial and bacterial inner membranes such as cardiolipin (CL). CG-MD simulations have been used to assess binding sites for CL with cytochrome bc_1_ [22] or cytochrome c oxidase [23^••^] and to estimate the free energy landscapes of these interactions [23^••^]. A comparable approach has been used to examine the interactions of cardiolipin with the ADP/ATP carrier ANT1 (Figure 2a). These studies confirm that such simulations can accurately reproduce lipid binding sites seen in the X-ray structure of this key mitochondrial transport proteins [1•, 7].

Simulations have also been applied to bacterial membranes [24] and their proteins. For example, combined structural, biophysical and computational studies have explored the role of lipids in the mechanosensitivity of the *Escherichia coli* ion channel MscS [25]. Selective interactions of CL with UraA, a bacterial inner membrane transporter, have also been explored [26]. Realistic modelling of the more complex outer membranes of Gram negative bacteria has required development of models for lipopolysaccharide (LPS), the major constituent of the outer leaflet of these membranes [27, 28]. Recent progress in both atomistic [29^•^] and coarse-grain [30] simulations of LPS enable studies of the interactions of a number of *E. coli* outer membrane proteins, for example FecA [31^••^], OmpLA [29^•^] and OmpF [32], with this complex membrane environment.

The nanoscale interactions of cytoplasmic peripheral membrane proteins and their lipid recognition domains with cell membranes may also be explored by simulations [33]. For example, MD simulations may be used to study how interactions with lipids such as PIPs may guide the recruitment of peripheral proteins such as PTEN to membranes within the cell [34], and also to explore the free energy landscapes [35^••^] underlying the interactions of lipid-recognition domains, such as PH domains [36] (Figure 2b), with PIP-containing membranes. The nanoscale effects of curvature and lipid composition on recruitment of peripheral proteins to cell membranes have also been explored by a combination of experiments and simulation [37^•^].

## Beyond the nanoscale: simulation of complex and crowded membrane systems

The diversity of lipids and proteins simulated and the accuracy with which interaction sites are identified, as surveyed in the previous section, demonstrate the efficacy of the simulation approach, and strengthens confidence in its extension to more complex membrane systems. Models can now incorporate the compositional complexity of cell membranes [38, 39•, 40], and mimic the crowding of proteins in cell membranes. Simulations of such models allow us to explore the emergent dynamics of complex and crowded membranes. In multi-component membranes lipids move in concert, with correlation times in the range of hundreds of nanoseconds, and correlation lengths of >10 nm [41], underlining the importance of large scale extended simulations to fully sample the interactions of the complex *in vivo* environment experienced by membrane proteins. These larger scale models help us to understand the collective behaviour of multiple copies of membrane proteins, such as the influence of crowding of membrane proteins on their clustering and diffusion [42, 43•]. These emergent dynamic properties of membranes may play a key role as regulatory mechanisms [44, 45], and will influence the mechanical properties of cell membranes.

Simulations have been used to explore lipid sorting and membrane (nano)domain formation. For example, long atomistic simulations have revealed substructures within ordered lipid phases, and have demonstrated coexistence of ordered and disordered lipid phases [46]. Large scale CG simulations have also provided insights into the degree of dynamic lateral heterogeneity as consequence of lipid clustering within models of mammalian cell membranes [38, 39•]. Protein clustering and oligomerization are observed within such large scale simulations. A number of studies have focussed on GPCR oligomerization and the influence of lipids. Thus, Periole *et al.* used CG-MD simulations to model supra-molecular assemblies of rhodopsin [47^•^]. Simulations of opioid receptors in a mixed POPC-cholesterol membrane helped to define the role of interfacial lipids at the protein-protein interface [48]. CG simulations of oligomerization of the β2-adrenergic receptor have explored the effects of protein-membrane hydrophobic mismatch [49]. Different mixtures of unsaturated and saturated lipids have been shown to affect the oligomerization of both adenosine and dopamine receptors [50]. Simulations of the sphingosine-1-phosphate receptor in a complex mixed-lipid asymmetric bilayer have revealed how protein–lipid–protein interactions may influence the dynamic clustering of GPCRs [51^•^].

**Figure 3:**
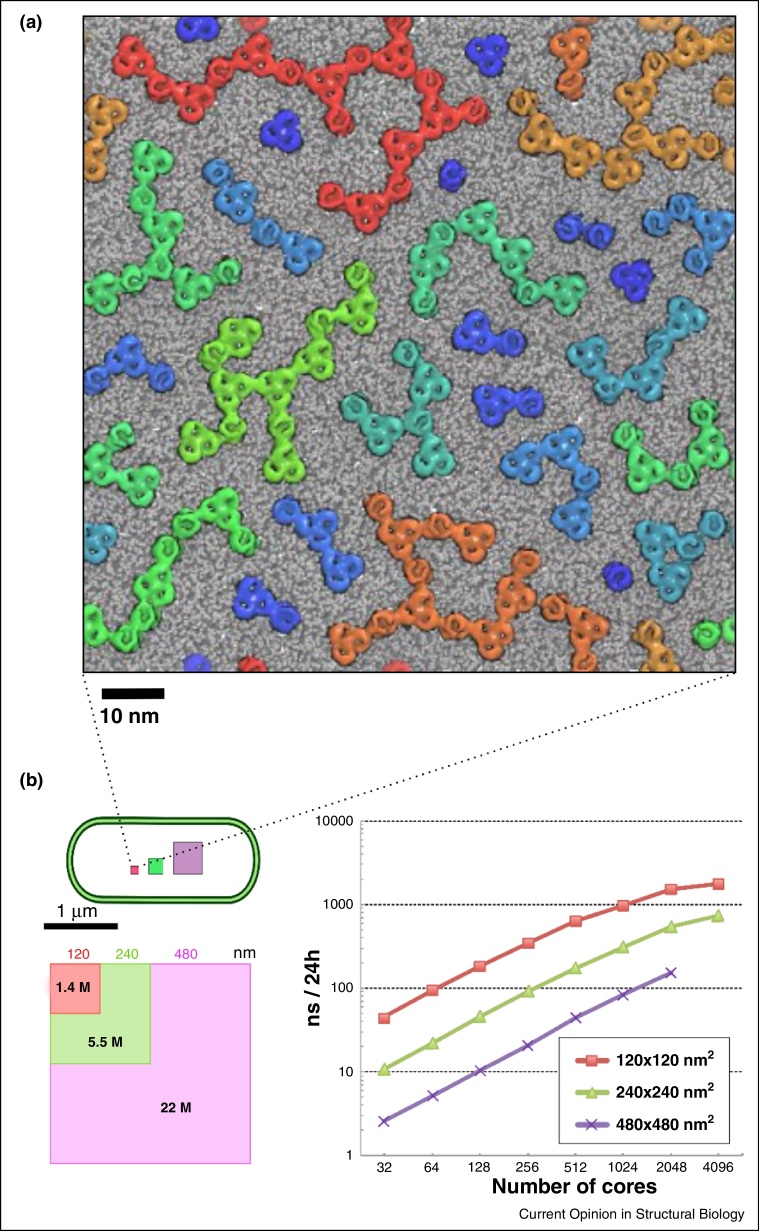
Large 2D membrane. **(a)** Snapshot from a CG simulation of 72 OmpF trimers and 72 BtuB molecules in a simple (PE/PG, in grey) lipid bilayer [54^•^]. The proteins are colour coded according to the size of the cluster of which they form part. (**b)** Schematic of system sizes for bacterial OMP simulations (left) showing approximate linear dimensions and number of particles, and scaling curves (right) for these system sizes, showing the number of nanoseconds simulated per day in relation to the number of CPUs on CURIE (http://www-hpc.cea.fr/en/complexe/tgcc-curie.htm).

This approach has been extended beyond the interactions of GPCRs. For example, simulations of a mitochondrial inner membrane indicate how cardiolipin may ‘glue’ together respiratory proteins into supercomplexes [52^•^]. Analysis of the free energy landscape of interaction of the bacterial outer membrane protein NanC has revealed how intervening lipids may stabilize a membrane protein dimer [53]. Such protein–lipid–protein interaction may underlie functionally important larger scale membrane organization. Thus, combining molecular dynamics simulations with *in vitro* and *in vivo* experimental studies has indicated how formation of large clusters of bacterial outer membrane proteins (OmpF and BtuB) may play a key role in the formation of membrane protein ‘islands’ during the division of bacterial cells [54^•^] (Figure 3a). The impact of protein clustering on membrane curvature has been demonstrated in a study of ATP synthase, combining electron cryotomography with simulations of ATP synthase dimers in a phospholipid bilayer [55].

Clustering has also been explored in larger scale simulations of peripheral membrane proteins. For example, clustering of lipid-anchored H-Ras has been observed in simulations of a 3 lipid component (di16:0 PC + di18:2 PC + cholesterol) bilayer, in which the protein accumulated at the interface between lipid ordered and lipid disordered regions, resulting in an increased local membrane curvature [56^•^]. CG simulations have also suggested that N-Ras clusters can alter the rate of formation of lipid phases in similar mixed lipid bilayers [57]. Other peripheral proteins may have dramatic effects on membrane properties. For example, simulations of α-synuclein aggregation [58] indicate how proteins may remodel the shapes of membranes, and large scale simulations of SNARE proteins suggest that hydrophobic mismatch may induce protein clustering and segregation [59].

## Approaching experimental length scales: large scale membrane simulations

Ongoing advances in for example the development of simulation codes to efficiently exploit very large scale computing resources, including CPU/GPU combination [60], and in methods for setup and analysis of complex simulation systems [61] enable molecular simulations of membranes to achieve length scales of several hundred nanometers, thus permitting direct comparison with cell membrane imaging by cryo-electron tomography and by superresolution optical microscopies. Using these approaches, simulations of for example whole virus particles and subcellular organelles become feasible. Furthermore, more highly coarse-grained (or mesoscopic) simulation approaches have been developed to aid modelling of emergent behaviours in these complex protein-membrane systems.

A landmark early study in this field is provided by a combined experimental and modelling study of synaptic vesicles [62], in which diverse data (from structural biology, mass spectroscopy, and biophysics) were integrated to develop a near atomic resolution model which could be compared to images from electron microscopy. With this proof of principle for developing such large scale models, it is now timely to embark on their simulation. For example, molecular simulations can enable dynamic structural models of enveloped viruses to be explored at atomic or near atomic resolution. Thus multiscale simulations, including a coarse-grained model of the lipid bilayer, have been used to the study the early stages of formation of the HIV capsid, [63^•^], whilst all-atom molecular dynamics have been used to fit a model of the mature HIV-1 capsid into cryo-electron microscopy density [64^••^]. CG-MD simulations have been used to probe the dynamic behaviour of lipid bilayer components of two viral envelopes: those of influenza A [65^••^] and of dengue virus [66]. In both cases simulations of the membrane envelopes of intact virions revealed slow and anomalous diffusion of the lipids, that is ‘raft-like’ behaviour of the viral membrane. Taken together these studies and others (reviewed in [67]) reveal considerable scope for the application of molecular simulations to viruses and their interactions with cell membranes. Other applications have explored large scale dynamic events including for example membrane fusion [68], and BAR domain-induced remodelling of vesicles [69, 70], including the influence of membrane tension on BAR assembly [71].

**Figure 4:**
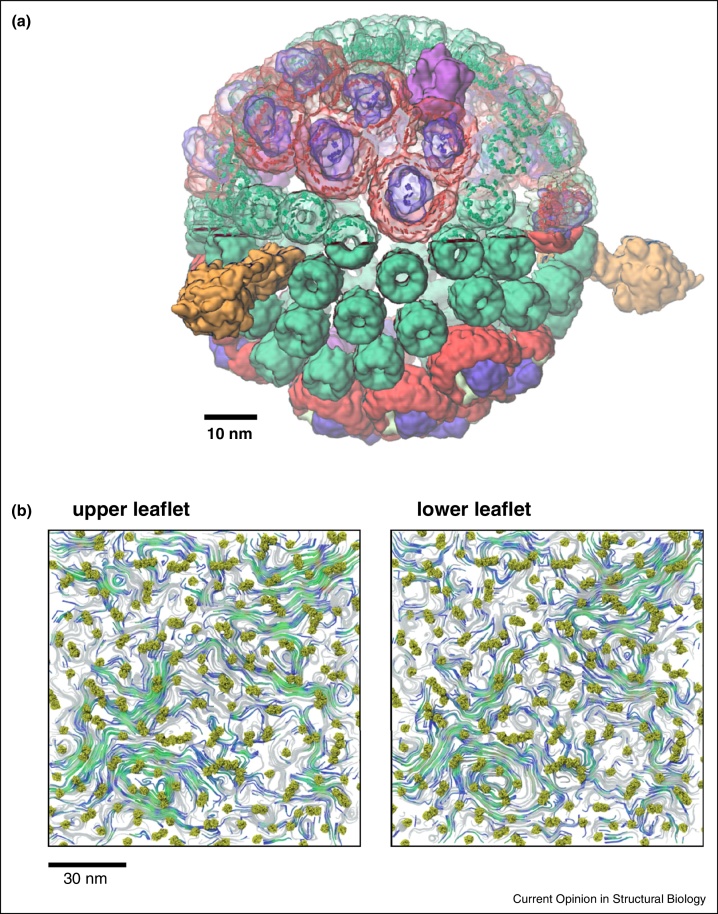
Very large systems and their visualization. **(a)** Model of a spherical chromatophore vesicle from *R. sphaeroides* ([72^••^], M Sener, unpublished data) (figure courtesy of Melih Sener, John Stone and Klaus Schulten). (**b)** Lipid flow visualization via streamlines, illustrated by a CG simulation of 256 OmpA proteins in a PE/PG bilayer [80]. The two panels correspond to the two leaflets of the lipid bilayer showing the protein (green) with streamlines illustrating the local lipid flow. Coloured streamlines depict correlated flows between the two leaflets, while the remaining streamlines are coloured in grey. Reproduced by permission of The Royal Society of Chemistry.

Molecular simulations in combination with AFM and spectroscopic data have been used to construct a model of an intact bacterial photosynthetic chromatophore (Figure 4a) ([72^••^], M Sener, unpublished data) enabling detailed modelling of excitation transfer between pigment molecule clusters. Highly coarse-grained (DPD) models have been used recently to study the dynamic organization of PSII–LHCII supercomplexes in plant photosynthetic membranes [73]. These and related models, which address the dynamic organization of thylakoid membranes on a several-hundred nanometer lengthscale, can be used to model light harvesting mechanisms, thus enabling direct comparison with spectroscopic data on these processes [74^••^].

The studies described above have made use of models over a range of scales, from atomistic to CG, building up to mesoscale simulations. Such investigations may be aided by simulation-based tools that allow for the melding of high resolution structural data with lower resolution data from for example cryo-EM [75]. With the recent advances in the resolution of cryo-EM and cryo-ET, and the current expansion in simulations carried out at close to experimental length scales, there is much opportunity for further development of mesoscale models.

Molecular simulations of complex membrane assemblies have benefitted from development of a range of tools for, for example, semi-automated setup of complex mixed lipid bilayer [76, 77]. On a larger scale for example cellPACK provides mesoscale packing algorithms to generate and visualize three-dimensional models of complex biological environments, and has been evaluated for models of synaptic vesicles and of an HIV virion [78]. Larger simulations also necessitate the use of significant computing resources (Figure 3b), and careful consideration of scaling on thousands of CPUs becomes important. The volume of data generated by large scale simulations is appreciable, in the range of hundreds of GB per simulation (Figure 1), which imposes substantial data storage and processing demands. Very large scale simulations also require development of novel methods for visualization (e.g. Quicksurf in VMD) [79] and for analysis of for example lipid flows in complex membranes (Figure 4b) [80]. It is clear that future developments are likely to further integrate a range of tools for setup, running, visualization and analysis of larger and more complex membrane systems, in addition to development of databases for storage and dissemination of the results of membrane simulations (e.g. MemProtMD [1^•^]).

## Conclusions

Using multiscale molecular simulations as a ‘computational microscope’ we can characterize the interactions of membrane proteins with lipid, matching, incorporating and extending the information which may be obtained from experimental structural and biophysical (e.g. MS) studies. Simulation approaches have been extended to allow crowded and complex membranes to be simulated with increasing biological realism. Having thus established the accuracy and utility of computational approaches to cell membranes, they are now being used to model and simulate cellular organelles and enveloped viruses. Paired with the growing wealth of cryo-EM and cryo-ET structural data, there is considerable promise for future ‘*in silico in vivo*’ studies of cell membranes.

## Conflict of interest

Nothing declared.

## References and recommended reading

Papers of particular interest, published within the period of review, have been highlighted as:

• of special interest
•• of outstanding interest

## References

[1•] Stansfeld P.J., Goose J.E., Caffrey M., Carpenter E.P., Parker J.L., Newstead S., Sansom M.S.P. (2015). MemProtMD: automated insertion of membrane protein structures into explicit lipid membranes. Structure.

[2] Stansfeld P.J., Sansom M.S.P. (2011). Molecular simulation approaches to membrane proteins. Structure.

[3] Laganowsky A., Reading E., Allison T.M., Ulmschneider M.B., Degiacomi M.T., Baldwin A.J., Robinson C.V. (2014). Membrane proteins bind lipids selectively to modulate their structure and function. Nature.

[4] Ingólfsson H.I., Arnarez C., Periole X., Marrink S.J. (2016). Computational “microscopy” of cellular membranes. J Cell Sci.

[5••] Marrink S.J., Tieleman D.P. (2013). Perspective on the Martini model. Chem Soc Rev.

[6] Shinoda W., DeVane R., Klein M.L. (2012). Computer simulation studies of self-assembling macromolecules. Curr Opin Struct Biol.

[7] Hedger G., Sansom M.S.P. (2016). Lipid interaction sites on channels, transporters and receptors: recent insights from molecular dynamics simulations. Biochim Biophys Acta.

[8] Pöyry S., Vattulainen I. (2016). Role of charged lipids in membrane structures — insight given by simulations. Biochim Biophys Acta.

[9] Aponte-Santamaría C., Briones R., Schenk A.D., Walz T., de Groot B.L. (2012). Molecular driving forces defining lipid positions around aquaporin-0. Proc Natl Acad Sci USA.

[10] Stansfeld P.J., Jefferys E.E., Sansom M.S.P. (2013). Multiscale simulations reveal conserved patterns of lipid interactions with aquaporins. Structure.

[11] Sengupta D., Chattopadhyay A. (2015). Molecular dynamics simulations of GPCR–cholesterol interaction: an emerging paradigm. Biochim Biophys Acta.

[12] Schmidt M.R., Stansfeld P.J., Tucker S.J., Sansom M.S.P. (2013). Simulation-based prediction of phosphatidylinositol 4,5-bisphosphate binding to an ion channel. Biochemistry.

[13] Kasimova M.A., Zaydman M.A., Cui J., Tarek M. (2015). PIP_2_-dependent coupling is prominent in Kv7.1 due to weakened interactions between S4-S5 and S6. Sci Rep.

[14•] Khelashvili G., Stanley N., Sahai M.A., Medina J., LeVine M.V., Shi L., De Fabritiis G., Weinstein H. (2015). Spontaneous inward opening of the dopamine transporter is triggered by PIP_2_-regulated dynamics of the N-terminus. ACS Chem Neurosci.

[15•] Hedger G., Sansom M.S.P., Koldsø H. (2015). The juxtamembrane regions of human receptor tyrosine kinases exhibit conserved interaction sites with anionic lipids. Sci Rep.

[16] Hall B.A., Halim K.B.A., Buyan A., Emmanouil B., Sansom M.S.P. (2014). Sidekick for membrane simulations: automated ensemble molecular dynamics simulations of transmembrane helices. J Chem Theory Comput.

[17] Wassenaar T.A., Pluhackova K., Moussatova A., Sengupta D., Marrink S.J., Tieleman D.P., Böckmann R.A. (2015). High-throughput simulations of dimer and trimer assembly of membrane proteins. The DAFT approach. J Chem Theory Comput.

[18] Hedger G., Koldsø H., Sansom M.S.P. (2016). Free energy landscape of lipid interactions with regulatory binding sites on the transmembrane domain of the EGF receptor. J Phys Chem B.

[19•] Arkhipov A., Shan Y., Kim E.T., Shaw D.E. (2014). Membrane interaction of bound ligands contributes to the negative binding cooperativity of the EGF receptor. PLoS Comput Biol.

[20••] Kaszuba K., Grzybek M., Orłowski A., Danne R., Róg T., Simons K., Coskun Ü., Vattulainen I. (2015). *N*-Glycosylation as determinant of epidermal growth factor receptor conformation in membranes. Proc Natl Acad Sci USA.

[21] Chavent M., Seiradake E., Jones E.Y., Sansom M.S.P. (2015). Structures of the EphA2 receptor at the membrane: role of lipid interactions. Structure.

[22] Arnarez C., Mazat J., Elezgaray J., Marrink S.J., Periole X. (2013). Evidence for cardiolipin binding sites on the membrane-exposed surface of the cytochrome bc_1_. J Am Chem Soc.

[23••] Arnarez C., Marrink S.J., Periole X. (2013). Identification of cardiolipin binding sites on cytochrome c oxidase at the entrance of proton channels. Sci Rep.

[24] Khakbaz P., Klauda J.B. (2015). Probing the importance of lipid diversity in cell membranes via molecular simulation. Chem Phys Lipids.

[25] Pliotas C., Dahl A.C.E., Rasmussen T., Mahendran K.R., Smith T.K., Marius P., Gault J., Banda T., Rasmussen A., Miller S. (2015). The role of lipids in mechanosensation. Nat Struct Mol Biol.

[26] Kalli A.C., Sansom M.S.P., Reithmeier R.a.F. (2015). Molecular dynamics simulations of the bacterial UraA H^+^-Uracil symporter in lipid bilayers reveal a closed state and a selective interaction with cardiolipin. PLoS Comput Biol.

[27] Parkin J., Chavent M., Khalid S. (2015). Molecular simulations of gram-negative bacterial membranes: a vignette of some recent successes. Biophys J.

[28] Pavlova A., Hwang H., Lundquist K., Balusek C., Gumbart J.C., Noskov S. (2016). Living on the edge: simulations of bacterial outer-membrane proteins. Biochim Biophys Acta.

[29•] Wu E.L., Fleming P.J., Yeom M.S., Widmalm G., Klauda J.B., Fleming K.G., Im W. (2014). *E. coli* outer membrane and interactions with OmpLA. Biophys J.

[30] Ma H., Irudayanathan F.J., Jiang W., Nangia S. (2015). Simulating gram-negative bacterial outer membrane: a coarse grain model. J Phys Chem B.

[31••] Piggot T.J., Holdbrook D.A., Khalid S. (2013). Conformational dynamics and membrane interactions of the *E. coli* outer membrane protein FecA: a molecular dynamics simulation study. Biochim Biophys Acta.

[32] Patel D.S., Re S., Wu E.L., Qi Y., Klebba P.E., Widmalm G., Yeom M.S., Sugita Y., Im W. (2016). Dynamics and interactions of OmpF and LPS: influence on pore accessibility and ion permeability. Biophys J.

[33] Kalli A.C., Sansom M.S.P. (2014). Interactions of peripheral proteins with model membranes as viewed by molecular dynamics simulations. Biochem Soc Trans.

[34] Kalli A.C., Devaney I., Sansom M.S.P. (2014). Interactions of PTEN proteins with phosphatidyl inositol phosphates: insights from molecular dynamics simulations of PTEN and VSP. Biochemistry.

[35••] Naughton F.B., Kalli A.C., Sansom M.S.P. (2016). Association of peripheral membrane proteins with membranes: free energy of binding of GRP1 PH domain with PIP-containing model bilayers. J Phys Chem Lett.

[36] Lai C.L., Srivastava A., Pilling C., Chase A.R., Falke J.J., Voth G.A. (2013). Molecular mechanism of membrane binding of the GRP1 PH domain. J Mol Biol.

[37•] Vanni S., Hirose H., Barelli H., Antonny B., Gautier R. (2014). A sub-nanometre view of how membrane curvature and composition modulate lipid packing and protein recruitment. Nat Commun.

[38] Koldsø H., Shorthouse D., Helie J., Sansom M.S.P. (2014). Lipid clustering correlates with membrane curvature as revealed by molecular simulations of complex lipid bilayers. PLoS Comput Biol.

[39•] Ingólfsson H.I., Melo M.N., Van Eerden F.J., Arnarez C., López C.A., Wassenaar T.A., Periole X., De Vries A.H., Tieleman D.P., Marrink S.J. (2014). Lipid organization of the plasma membrane. J Am Chem Soc.

[40] van Eerden F.J., de Jong D.H., de Vries A.H., Wassenaar T.A., Marrink S.J. (2015). Characterization of thylakoid lipid membranes from cyanobacteria and higher plants by molecular dynamics simulations. Biochim Biophys Acta.

[41] Apajalahti T., Niemel P., Govindan P.N., Miettinen M.S., Salonen E., Marrink S.J., Vattulainen I. (2010). Concerted diffusion of lipids in raft-like membranes. Faraday Discuss.

[42] Javanainen M., Hammaren H., Monticelli L., Jeon J.-H., Miettinen M.S., Martinez-Seara H., Metzler R., Vattulainen I. (2013). Anomalous and normal diffusion of proteins and lipids in crowded lipid membranes. Faraday Discuss.

[43•] Goose J.E., Sansom M.S.P. (2013). Reduced lateral mobility of lipids and proteins in crowded membranes. PLoS Comput Biol.

[44] Iversen L., Mathiasen S., Larsen J.B., Stamou D. (2015). Membrane curvature bends the laws of physics and chemistry. Nat Chem Biol.

[45] Guigas G., Weiss M. (2015). Effects of protein crowding on membrane systems. Biochim Biophys Acta.

[46] Sodt A.J., Sandar M.L., Gawrisch K., Pastor R.W., Lyman E. (2014). The molecular structure of the liquid-ordered phase of lipid bilayers. J Am Chem Soc.

[47•] Periole X., Knepp A.M., Sakmar T.P., Marrink S.J., Huber T. (2012). Structural determinants of the supramolecular organization of G protein-coupled receptors in bilayers. J Am Chem Soc.

[48] Provasi D., Boz M.B., Johnston J.M., Filizola M. (2015). Preferred supramolecular organization and dimer interfaces of opioid receptors from simulated self-association. PLoS Comput Biol.

[49] Mondal S., Johnston J.M., Wang H., Khelashvili G., Filizola M., Weinstein H. (2013). Membrane driven spatial organization of GPCRs. Sci Rep.

[50] Guixà-González R., Javanainen M., Gómez-Soler M., Cordobilla B., Domingo J.C., Sanz F., Pastor M., Ciruela F., Martinez-Seara H., Selent J. (2016). Membrane omega-3 fatty acids modulate the oligomerisation kinetics of adenosine A_2A_ and dopamine D_2_ receptors. Sci Rep.

[51•] Koldsø H., Sansom M.S.P. (2015). Organization and dynamics of receptor proteins in a plasma membrane. J Am Chem Soc.

[52•] Arnarez C., Marrink S.J., Periole X. (2016). Molecular mechanism of cardiolipin-mediated assembly of respiratory chain supercomplexes. Chem Sci.

[53] Dunton T.A., Goose J.E., Gavaghan D.J., Sansom M.S.P., Osborne J.M. (2014). The free energy landscape of dimerization of a membrane protein, NanC. PLoS Comput Biol.

[54•] Rassam P., Copeland N.A., Birkholz O., Tóth C., Chavent M., Duncan A.L., Cross S.J., Housden N.G., Kaminska R., Seger U. (2015). Supramolecular assemblies underpin turnover of outer membrane proteins in bacteria. Nature.

[55] Davies K.M., Anselmi C., Wittig I., Faraldo-Gómez J.D., Kühlbrandt W. (2012). Structure of the yeast F1Fo-ATP synthase dimer and its role in shaping the mitochondrial cristae. Proc Natl Acad Sci USA.

[56•] Janosi L., Li Z., Hancock J.F., Gorfe A.A. (2012). Organization, dynamics, and segregation of Ras nanoclusters in membrane domains. Proc Natl Acad Sci USA.

[57] Jefferys E., Sansom M.S.P., Fowler P.W. (2014). NRas slows the rate at which a model lipid bilayer phase separates. Faraday Discuss.

[58] Braun A.R., Lacy M.M., Ducas V.C., Rhoades E., Sachs J.N. (2014). α-Synuclein-induced membrane remodeling is driven by binding affinity, partition depth, and interleaflet order asymmetry. J Am Chem Soc.

[59] Milovanovic D., Honigmann A., Koike S., Göttfert F., Pähler G., Junius M., Müllar S., Diederichsen U., Janshoff A., Grubmüller H. (2015). Hydrophobic mismatch sorts SNARE proteins into distinct membrane domains. Nat Commun.

[60] Kutzner C., Páll S., Fechner M., Esztermann A., De Groot B.L., Grubmüller H. (2015). Best bang for your buck: GPU nodes for GROMACS biomolecular simulations. J Comput Chem.

[61] Pronk S., Pouya I., Lundborg M., Rotsko G., Kasson P.M., Lindahl E. (2015). Molecular simulation workflows as parallel algorithms: the execution engine of Copernicus, a distributed high-performance computing platform. J Chem Theory Comput.

[62] Takamori S., Holt M., Stenius K., Lemke E.A., Grønborg M., Riedel D., Urlaub H., Schenck S., Brügger B., Ringler P. (2006). Molecular anatomy of a trafficking organelle. Cell.

[63•] Grime J.M.A., Voth G.A. (2012). Early stages of the HIV-1 capsid protein lattice formation. Biophys J.

[64••] Zhao G., Perilla J.R., Yufenyuy E.L., Meng X., Chen B., Ning J., Ahn J., Gronenborn A.M., Schulten K., Aiken C. (2013). Mature HIV-1 capsid structure by cryo-electron microscopy and all-atom molecular dynamics. Nature.

[65••] Reddy T., Shorthouse D., Parton D.L., Jefferys E., Fowler P.W., Chavent M., Baaden M., Sansom M.S.P. (2015). Nothing to sneeze at: a dynamic and integrative computational model of an influenza A virion. Structure.

[66] Reddy T., Sansom M.S.P. (2016). The role of the membrane in the structure and biophysical robustness of the dengue virion envelope. Structure.

[67] Reddy T., Sansom M.S.P. (2016). Computational virology: from the inside out. Biochim Biophys Acta.

[68] Risselada H.J., Smirnova Y., Grubmüller H. (2014). Free energy landscape of rim-pore expansion in membrane fusion. Biophys J.

[69] Yu H., Schulten K. (2013). Membrane sculpting by F-BAR domains studied by molecular dynamics simulations. PLoS Comput Biol.

[70] Simunovic M., Voth G.A., Callan-Jones A., Bassereau P. (2015). When physics takes over: BAR proteins and membrane curvature. Trends Cell Biol.

[71] Simunovic M., Voth G.A. (2015). Membrane tension controls the assembly of curvature-generating proteins. Nat Commun.

[72••] Sener M.K., Olsen J.D., Hunter C.N., Schulten K. (2007). Atomic-level structural and functional model of a bacterial photosynthetic membrane vesicle. Proc Natl Acad Sci USA.

[73] Lee C.-K., Pao C.-W., Smit B. (2015). PSII-LHCII supercomplex organizations in photosynthetic membrane by coarse-grained simulation. J Phys Chem B.

[74••] Amarnath K., Bennett D.I.G., Schneider A.R., Fleming G.R. (2015). Mechanisms of light harvesting by photosystem II in plants. Proc Natl Acad Sci USA.

[75] McGreevy R., Teo I., Singharoy A., Schulten K. (2016). Advances in the molecular dynamics flexible fitting method for cryo-EM modeling. Methods.

[76] Wu E.L., Cheng X., Jo S., Rui H., Song K.C., Dávila-Contreras E.M., Qi Y., Lee J., Monje-Galvan V., Venable R.M. (2014). CHARMM-GUI *Membrane Builder* toward realistic biological membrane simulations. J Comput Chem.

[77] Wassenaar T.A., Ingólfsson H.I., Böckmann R.A., Tieleman D.P., Marrink S.J. (2015). Computational lipidomics with insane: a versatile tool for generating custom membranes for molecular simulations. J Chem Theory Comput.

[78] Johnson G.T., Autin L., Al-Alusi M., Goodsell D.S., Sanner M.F., Olson A.J. (2014). cellPACK: a virtual mesoscope to model and visualize structural systems biology. Nat Methods.

[79] Perilla J.R., Goh B.C., Stone J., Schulten K. (2015). Chemical visualization of human pathogens: the retroviral capsids. Proceedings of the 2015 ACM/IEEE Conference on Supercomputing.

[80] Chavent M., Reddy T., Goose J., Dahl A.C.E., Stone J.E., Jobard B., Sansom M.S.P. (2014). Methodologies for the analysis of instantaneous lipid diffusion in MD simulations of large membrane systems. Faraday Discuss.

